# Smoke Nuisance
*Prize Essay on the Prevention of the Smoke Nuisance. By C. W. Williams, C.E. London; John Weale. 1856.


**Published:** 1856-10

**Authors:** 


### THE EPITOME OF SANITARY LITERATURE.

### SMOKE NUISANCE.*

Mr. Williams's Prize Essay, on *The Prevention of the Smoke Nuisance,* will repay perusal. He argues that the "combustion of smoke", according to the common acceptation of the term, is impossible. He traces the carbon rising from a fire through four stages: 1. As carburetted hydrogen gas; 2. As raised by heat to the temperature of incandescence; 3. As confirmed with oxygen as invisible carbonic acid; 4. As lamp black or soot, having escaped combustion by not having had access to air before it was cooled below the temperature required for chemical action. The remedy for smoke, therefore, is, to provide for the admission of air to the furnace at the proper time and in proper quantity, so as to insure the combustion of the carburetted hydrogen gas, at the time when, from its high temperature of incandescence, it is best fitted to receive it.

* Prize Essay on the Prevention of the Smoke Nuisance. By C. W. Williams, C.E. London; John Weale. 1856.

